# Lower pneumococcal IgG levels and respiratory hospitalisation in children with Down syndrome; an exploratory study

**DOI:** 10.1007/s00431-026-07364-w

**Published:** 2026-08-28

**Authors:** Juliette Krop, Noortje B. Eijsvoogel, Jean-Luc Murk, Marco W. J. Schreurs, Gijs Th. J. van Well, Levinus A. Bok, Esther de Vries

**Affiliations:** 1 Tranzo, Tilburg School of Social and Behavioral Sciences, Tilburg University, PO Box 90153 (RP219), Tilburg, 5000LE the Netherlands; 2 Laboratory of Medical Microbiology and Immunology (Microvida), St. Elisabeth Hospital (ETZ), Tilburg, the Netherlands; 3 Department of Paediatrics, Maastricht University Medical Centre, Maastricht, the Netherlands; 4 Department of Paediatrics, Máxima Medical Centre, Veldhoven, the Netherlands; 5 Jeroen Bosch Academy Research, Jeroen Bosch Hospital, ‘S-Hertogenbosch, the Netherlands

**Keywords:** Down syndrome, Pneumococcal IgG concentration, Respiratory infection, Immune deficiency, ELISA, Luminex

## Abstract

Children with Down syndrome (DS) are at increased risk for recurrent and severe respiratory infections. Pneumococcal IgG measurements are used in clinical practice to evaluate humoral immunity, yet their clinical relevance in DS remains unclear. We examined potential associations between pneumococcal IgG concentrations and clinical characteristics in children with DS, including recurrent infections and respiratory hospital admission. We conducted a retrospective study in a paediatric DS cohort with repeated pneumococcal IgG measurements obtained from left-over plasma samples using ELISA and multiplex bead-based (Luminex) assays. Potential associations with selected clinical variables, including respiratory hospital admission, the presence of autoimmune comorbidity, and infections, were assessed using mixed-effects modelling. A cross-sectional analysis using only the last available measurement per participant was performed to address the temporal uncertainty between the static information on the clinical variables and the repeated antibody measurements. Across both assays, a history of hospital admission for respiratory problems was associated with lower pneumococcal IgG concentrations in linear mixed-effect regression analyses; a history of autoimmune comorbidity was associated with higher concentrations. A history of recurrent ear infection was associated with lower ELISA-measured IgG concentrations in the last-timepoint analysis. *Conclusion*: Our exploratory analysis suggests a potential association between lower pneumococcal IgG concentration and selected clinical characteristics in children with DS. These findings support further investigation into the clinical relevance of pneumococcal antibody measurements in children with DS in larger, prospective studies.

**Table:** 

**What is Known:** *• Down syndrome is associated with increased respiratory infections* *• Pneumococcal IgG measurements can be used to assess humoral immune function*	
**What is New:** *• Lower pneumococcal IgG concentrations were observed in the children with a history of respiratory hospital admission* *• Higher pneumococcal IgG concentrations were observed in the children with a history of autoimmunity*

**Supplementary information:**

The online version contains supplementary material available at https://doi.org/10.1007/s00431-026-07364-w.

## Introduction

Down syndrome (DS) is one of the most common chromosomal abnormalities in live-born infants [1]. Individuals with DS face a broad spectrum of health challenges, including cognitive impairment, congenital anomalies, and immunological dysfunction. Among these, an increased susceptibility to infections, particularly recurrent respiratory tract infections (RTIs) is an important clinical concern [2, 3] as it contributes to both increased morbidity and mortality [4]. This increased susceptibility to respiratory tract infections in DS is multifactorial and reflects not only immunological abnormalities, but also structural and functional upper and lower airway factors [5]. These include hypotonia, upper airway abnormalities, impaired airway clearance, aspiration, sleep-disordered breathing, and congenital heart disease, all of which may contribute to respiratory morbidity in this population [4, 6]. Most RTIs are likely viral in origin, although bacterial infections, particularly those caused by encapsulated organisms such as *Streptococcus pneumoniae*, also occur [7].

Previous studies have documented various immunological impairments in DS, including abnormalities in humoral immunity, cellular immunity, and phagocyte function [3, 8–13]. Children with DS typically exhibit a mildly to moderately reduced T- and B-cell count, and the function of these cells is also slightly impaired [3, 10–14]. However, a direct correlation between B-cell counts and clinical outcomes such as hospitalisation rates has not been consistently found [4, 11]. While previous work has demonstrated that children with DS can generate pneumococcal antibodies following vaccination, concentrations are often lower than in age-matched controls [15, 16]. Other vaccine-induced antibody responses in individuals with DS generally show lower antibody concentrations in the DS population but above the concentration considered protective [17–19]. These findings suggest that antibody concentrations might serve as a useful marker of functional humoral immunity in DS.

Recently, we showed that a 23-valent pneumococcal ELISA concentration moderately correlates with serotype-specific concentrations from multiplex bead-based (Luminex) assays [20]. In connection with this, we aimed to explore whether pneumococcal antibody concentrations, measured via both ELISA and Luminex, could be associated with clinical characteristics in children with DS (e.g., hospitalisation for respiratory problems, recurrent infections). Although this study was not designed to classify individual patients, identifying group-level associations may help to improve understanding of how pneumococcal antibody concentrations might relate to clinical characteristics in children with DS.

## Materials and methods

### Study design and samples

This retrospective cohort study was conducted at the Máxima Medical Centre (MMC) in Veldhoven and at the Laboratory for Medical Microbiology and Immunology (LMMI, Microvida) at the Elisabeth-TweeSteden Hospital (ETZ) in Tilburg, the Netherlands. Parents of children with DS (or the children themselves, if possible, when over 12 years of age) were approached for informed consent to use stored plasma samples that were no longer needed. These leftover samples have been collected during routine care in children visiting the yearly outpatient follow-up DS clinic over the past 15 years and stored at −80 °C in the laboratory department at MMC.

Information letters and informed consent forms were sent to the last known home address. If no response was received, a reminder was sent after two months. Once informed consent was obtained, samples were pseudonymised at the MMC before transportation on dry ice to the LMMI for immunological analysis. In some cases, the first plasma sample timepoint available was in early adulthood (*n* = 4). Each child could have up to seven plasma samples collected at various ages, ranging from 6 months to over 246 months (20 years of age). In total, 212 plasma samples from 62 individuals could be included in the study. The retrospective clinical data were extracted manually from the MMC electronic health records (EHRs) by author NBE and recorded as binary variables. These included static patient characteristics, such as sex, and congenital heart disease; recent clinical variables referring to the period since the previous blood draw or the year prior to sampling, such as recent infections; and cumulative clinical variables reflecting events occurring over the broader clinical history, including vaccination history, hospital admissions for respiratory problems, inhalation medication, antibiotic prophylaxis and therapeutic antibiotic use, recurrent ear infections, recurrent urinary tract infections (UTIs), and autoimmunity. Autoimmunity was defined as the presence of autoimmune comorbidities, including autoimmune diabetes mellitus, hypothyroidism, and coeliac disease. Due to the low incidence of these conditions individually, they were combined into the single composite variable ‘autoimmunity’ to ensure sufficient statistical power. Due to the retrospective nature of the study, spanning almost two decades, not enough details were available to assign more specific respiratory diagnoses to the hospital admissions. The proportion of missing data for all clinical variables is shown in Supplementary Table 1.

### Pneumococcal IgG concentrations

Total pneumococcal IgG concentrations were measured using the VaccZyme™ anti-PCP IgG ELISA kit (The Binding Site, Birmingham, United Kingdom) with pre-coated microtiter plates, following the manufacturer’s protocol, targeting 23 pneumococcal serotypes: 1, 2, 3, 4, 5, 6B, 7 F, 8, 9 N, 9 V, 10 A, 11 A, 12 F, 14, 15B, 17 F, 18 C, 19 A, 19 F, 20, 22 F, 23 F, 33F. The ELISA protocol included cell wall polysaccharide (CPS) absorption to mitigate non-specific binding [20].

Serotype-specific IgG concentrations were measured using an in-house developed Luminex-based multiplex immunoassay (MAGPIX, Luminex Corporation, Austin, TX, USA), targeting the same 23 serotypes and also including CPS absorption, as previously described [21, 22]. Both immunological assays were performed at the LMMI laboratory in Tilburg. For two samples, there was only enough material to perform the Luminex assay, and not the ELISA assay as well.

### Data preparation and statistical analysis

All data were analysed using R version 4.4.2 (Windows). To compare the techniques, we used the cumulative Luminex concentrations (sum of all 23-serotypes) since the ELISA assay measures all 23 serotypes together, also resulting in a sum concentration.

Outlier detection was performed using Cook’s distance and Mahalanobis distance on both raw and log-transformed concentrations. Consistent outliers, meaning in both Mahalanobis and Cook’s distance as well as raw and log data, were removed from the downstream analysis. Outlier diagnostics were conducted separately for ELISA and Luminex measurements (one-dimensional analyses) to identify assay-specific extreme values. In addition, a two-dimensional Mahalanobis and Cook’s distance was calculated on paired ELISA and Luminex measurements to identify samples showing discordance between assays. One sample was identified as a consistent two-dimensional discordant outlier across all diagnostic approaches and was excluded from all subsequent analyses. No consistent assay-specific outliers were identified for ELISA-only, while one Luminex-specific outlier was excluded from Luminex-only analyses (Supplementary Fig. 1).

Both raw and log10-transformed ELISA and Luminex concentrations were assessed. Visual inspection (histograms, QQ plots) and Shapiro–Wilk tests showed that log-transformation improved normality. Therefore, log-transformed concentrations were used for all subsequent analyses (Supplementary Fig. 2).

To compare the total ELISA concentration with the cumulative Luminex concentration, we computed intraclass correlation coefficients (ICCs) using a two-way model for consistency. ICCs were calculated after removal of the identified outlier. Agreement was classified as poor (< 0.5), moderate (0.5–0.75), good (0.75–0.9), or excellent (> 0.9).

### Modelling approaches: LMER and LM

We explored whether clinical variables or their interactions might be associated with log-transformed ELISA concentrations and log-transformed cumulative Luminex concentrations. Two modelling strategies were applied separately, one for the ELISA and one for the Luminex data. First, linear mixed-effects models (LMER) were fitted using all timepoints per individual (up to seven), with a random intercept for each participant to account for intra-individual correlations. Second, as explained above, a standard linear regression (LM) was applied using only the final available measurement per participant as a last-timepoint analysis; this cross-sectional approach helped reduce bias due to time-invariant clinical variables (e.g., clinical data that were the same across time).

The base model in both approaches included: recurrent ear infections, respiratory hospital admission, antibiotic prophylaxis, therapeutic antibiotic use, autoimmunity, inhalation medication, recent infections (in the year prior to drawing the sample), congenital heart disease and sex. Prior to model fitting, correlations between clinical variables, age at sampling, vaccination status, and antibody titers were explored using a correlation matrix. Pairwise Spearman correlations are presented in Supplementary Fig. 3. Age at blood draw and vaccination status showed substantial overlap with each other. Including these variables in the same model reduced interpretability of individual effect estimates and increased the risk of multicollinearity. Therefore, age and vaccination status were excluded from the final regression models. However, because age is biologically relevant to pneumococcal antibody concentrations we performed additional exploratory analyses. In these exploratory analyses, mixed-effects models incorporating age at first sampling and within-participant age increase between samples were evaluated to assess whether the longitudinal age structure of the dataset influenced the original findings. Both random-intercept and random-slope specifications were explored. As inclusion of a random slope did not meaningfully improve model performance, the final analyses were based on random-intercept models only. Results of these additional analyses are presented in Supplementary Tables 2, 3, and 4. Recurrent UTI was excluded because of limited clinical relevance.

Model building followed a stepwise, information-theoretic approach. Each candidate variable was first evaluated individually in a mixed-effects model with a random intercept for subject and compared to an intercept-only null model using the Akaike Information Criterion (AIC). Variables that reduced AIC relative to the intercept-only model were considered indicators of improved model fit and were retained for construction of assay-specific baseline multivariable models. All mixed-effects models were fitted using restricted maximum likelihood (REML). Model comparisons based on AIC were used descriptively to explore relative model fit. Missing values were encoded as NA and excluded. An overview of missing data per variable is provided in Supplementary Table 1.

To enable consistent interaction analyses across assays, a harmonised core set of variables that improved model fit across both assays was defined, supplemented with recurrent ear infections based on a priori clinical relevance. These core models were subsequently used as the reference framework for interaction testing.

### Interaction selection procedures

To investigate whether specific combinations of clinical variables affected model fit, interaction terms were explored. First, an interaction screening was performed in which each two-way interaction between main effects was added individually to the base model. Effect estimates, *p*-values, and AIC values were recorded. Next, a forward selection procedure was applied, where interactions were sequentially added if model fit improved based on a decrease in the AIC. A backward elimination procedure using the drop1() function was also tested, starting from the full model including all interactions and iteratively removing interactions with high *p*-values. Finally, an automated backward stepwise elimination was performed, removing interactions with Pr(> F) values exceeding 0.10, while monitoring AIC at each step. No interaction consistently improved model fit or reached statistical significance across these methods.

### Final regression analysis on single timepoint

To assess whether any of the clinical variables might be associated with ELISA or Luminex concentration at the most recent timepoint, we constructed a linear model (LM) using the last available measurement per participant. This way we ensured that the clinical variables (which did not vary over time) aligned better with the outcome variable. A generalised linear model (GLM) with Gaussian family was also tested and yielded similar results (data not shown).

## Results

The study cohort consisted of 62 children with DS contributing a total of 212 plasma samples. Most participants contributed repeated measurements over time, with a median of 2 samples per participant (range 1–7), and 51 participants (82.3%) had more than one sample available for analysis. Ages at sample collection ranged from 6 to 246 months. Baseline cohort characteristics are summarised in Table 1, and the distribution of repeated measurements per participant is shown in Supplementary Table 5.

**Table 1:** Participant characteristics

*Characteristic*	*Total cohort*
*Participants, n*	62
*Plasma samples, n*	212
*Male sex, n (%)*	34 (54,8)
*Age at sample collection, range*	6–246 months (0.5–20.5 years)
*Median samples per participant (range)*	2 (1–7)
*Participants with* > *1 sample, n (%)*	51 (82,3)
*Congenital heart disease, n (%)*	29 (46,8)
*Respiratory hospital admission, n (%)*	22 (35,5)
*Autoimmunity, n (%)*	31 (50)
*Recurrent ear infections, n (%)*	40 (64,5)
*Antibiotic prophylaxis, n (%)*	34 (54,8)
*Therapeutic antibiotic use, n (%)*	36 (58,1)
*Infections in past year*	26 (41,9)
*Inhalation medication*	13 (21,0)

### ELISA and Luminex

**Fig. 1:**
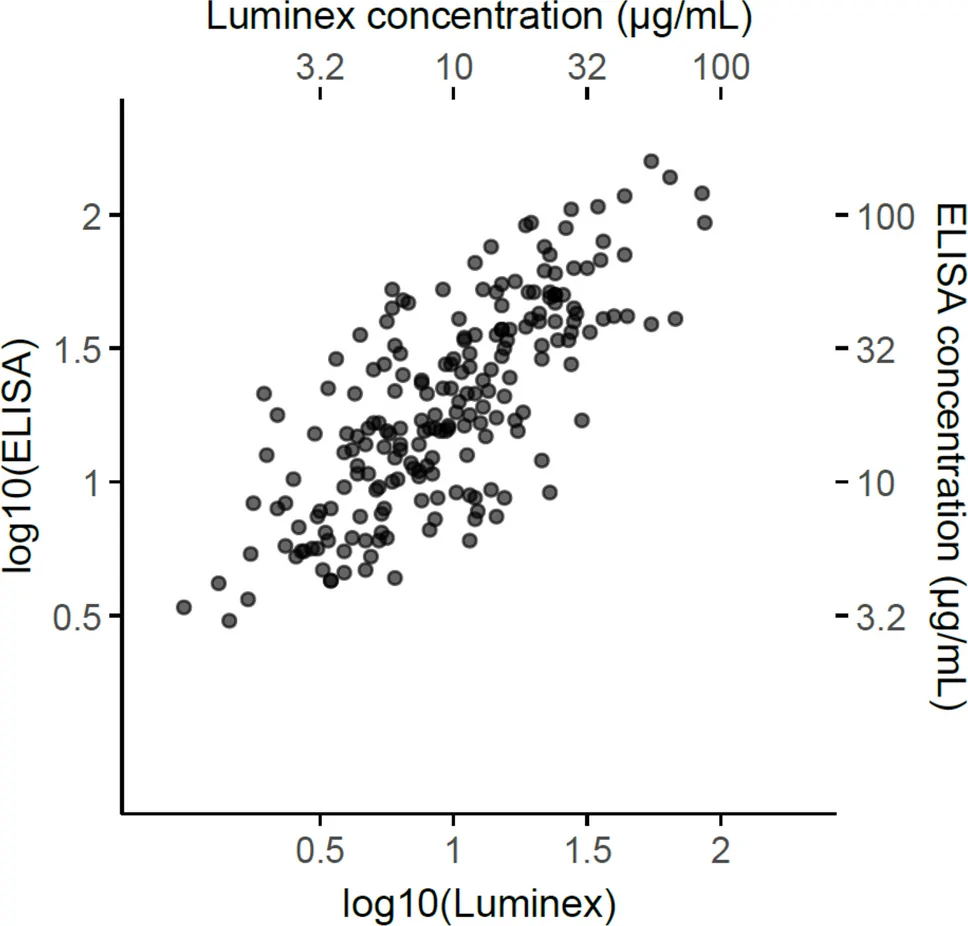
Agreement between ELISA- and Luminex-based measurements of pneumococcal IgG concentration. Each point represents a paired log10-transformed concentration obtained from the same sample (*n* = 209). ELISA reflects total 23-valent pneumococcal IgG, whereas Luminex represents the summed concentrations of the 23 individual serotypes. The intraclass correlation coefficient (ICC, two-way consistency model) was 0.76 (95% CI 0.69–0.81)

We first assessed the correlation between the 23-valent pneumococcal IgG concentrations measured via ELISA and the cumulative Luminex concentrations. The one assay-discordant outlier, one Luminex-specific outlier (Supplementary Fig. 1), and two samples without paired ELISA measurements were excluded. Following these exclusions, the log-transformed concentrations showed moderate agreement, with an ICC of 0.76 (95% CI: 0.69–0.81), indicating good reliability between the two measurement techniques in the cohort (Fig. 1).

### Linear Mixed-Effects Modelling (LMER)

A baseline LMER model was fitted using all available measurements, with a random intercept for each participant to account for the repeated observations. Candidate clinical variables were first evaluated individually by comparing each one-variable model against the intercept-only model using the AIC (Tables 2 and 3).

**Table 2:** Exploratory one-variable mixed-effects models for ELISA-based pneumococcal IgG log10 concentrations

*Variable*	*Estimate*	*95% CI*	*AIC*	*ΔAIC*
*Intercept only*			133.16	0.00
*Admission for respiratory problems*	− 0.16	− 0.34 to 0.02	119.53	− 13.63
*Therapeutic antibiotic use*	− 0.07	− 0.27 to 0.12	121.45	− 11.70
*Autoimmunity*	0.21	0.05 to 0.37	131.85	− 1.31
*Antibiotic prophylaxis*	− 0.19	− 0.35 to − 0.02	133.24	0.07
*Sex*	0.14	− 0.03 to 0.30	135.61	2.44
*Infections (prior year)*	− 0.13	− 0.30 to 0.03	135.76	2.59
*Recurrent ear infections*	− 0.12	− 0.30 to 0.5	136.15	2.98
*Inhalation medication*	0.03	− 0.18 to 0.23	137.79	4.63
*Congenital heart disease*	0.04	− 0.12 to 0.21	138.00	4.84

Estimates represent the estimated difference in log10 pneumococcal IgG concentration between participants with and without the indicated clinical variable in individual mixed-effects models. Negative estimates indicate lower pneumococcal IgG concentrations associated with the variable. ΔAIC values below 0 indicate improved model fit relative to the intercept-only model

*CI* Confidence interval, *AIC* Akaike Information Criterion

**Table 3:** Exploratory one-variable mixed-effects models for Luminex-based pneumococcal IgG log10 concentrations

*Variable*	*Estimate*	*95% CI*	*AIC*	*ΔAIC*
*Intercept only*			92.12	0.00
*Admission for respiratory problems*	− 0.14	− 0.33 to 0.06	88.18	− 3.93
*Therapeutic antibiotic use*	− 0.06	− 0.28 to 0.15	97.56	5.44
*Autoimmunity*	0.20	0.04 to 0.37	91.47	− 0.65
*Antibiotic prophylaxis*	− 0.19	− 0.36 to − 0.02	92.40	0.28
*Sex*	0.12	− 0.05 to 0.30	95.09	2.97
*Infections (prior year)*	− 0.15	− 0.33 to 0.02	93.99	1.87
*Recurrent ear infections*	− 0.00	− 0.17 to 0.18	97.06	4.94
*Inhalation medication*	− 0.10	− 0.31 to 0.12	95.93	3.81
*Congenital heart disease*	0.10	− 0.08 to 0.27	95.93	3.81

Estimates represent the estimated difference in log10 pneumococcal IgG concentration between participants with and without the indicated clinical variable in individual mixed-effects models. Negative estimates indicate lower pneumococcal IgG concentrations associated with the variable. ΔAIC values below 0 indicate improved model fit relative to the intercept-only model

*CI* Confidence interval, *AIC* Akaike Information Criterion

In the ELISA-based analyses, respiratory hospital admission showed the largest improvement in model fit (ΔAIC =  − 13.63), followed by *therapeutic* antibiotic use (ΔAIC =  − 11.71) and autoimmunity (ΔAIC =  − 1.31) (Table 2). Whereas antibiotic *prophylaxis*, sex, recent infections within the prior year, recurrent ear infections, inhalation medication, and congenital heart disease did not improve model fit relative to the intercept-only model (ΔAIC > 0 for each).

In the Luminex-based analyses, respiratory hospital admission again showed the strongest improvement in model fit (ΔAIC =  − 3.93), with a smaller improvement observed for autoimmunity (ΔAIC =  − 0.65) (Table 3). Antibiotic prophylaxis, recent infections, sex, inhalation medication, recurrent ear infections, therapeutic antibiotic use, and congenital heart disease did not improve model fit (ΔAIC > 0 for each).

**Fig. 2:**
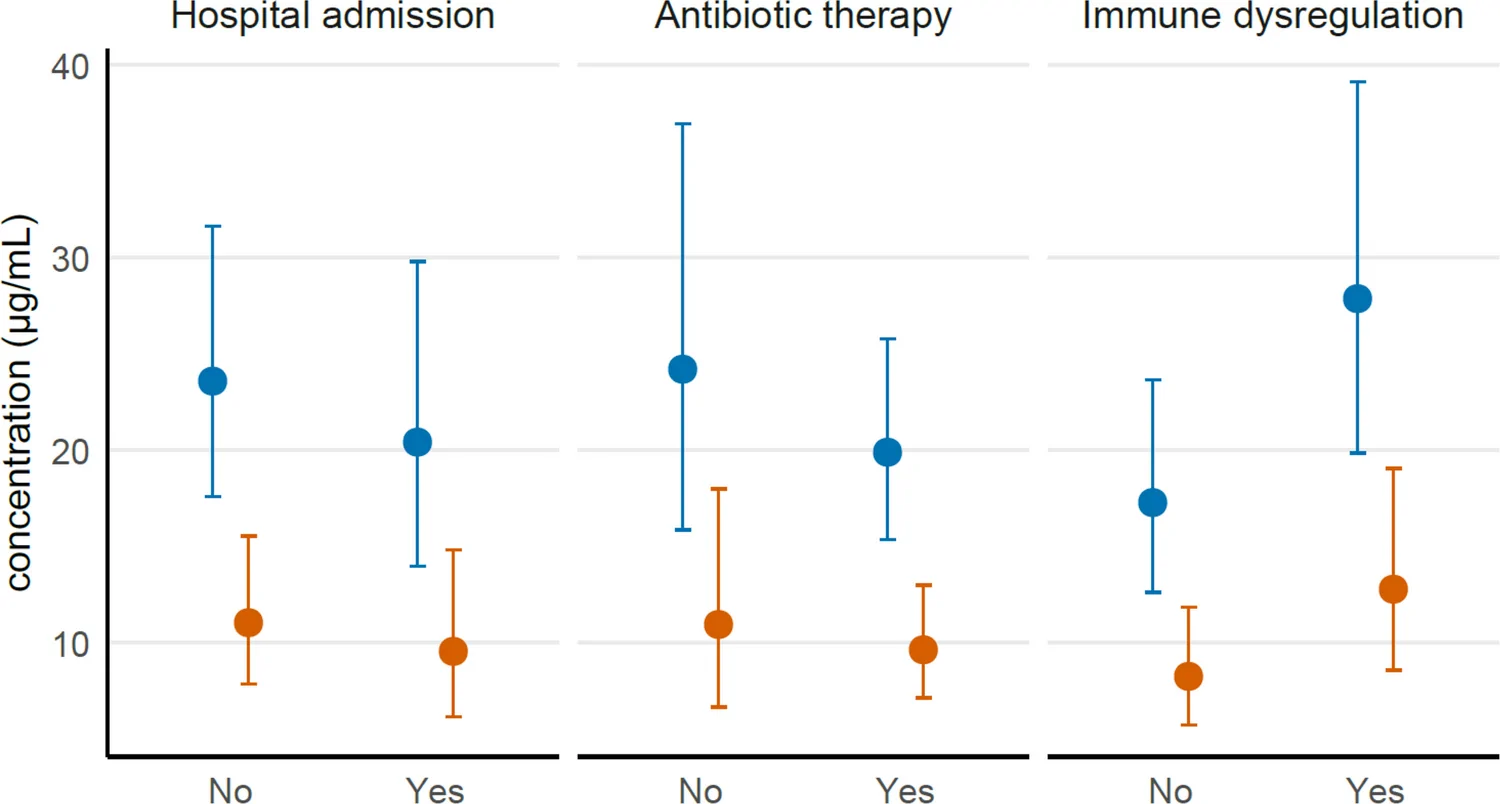
Predicted pneumococcal IgG concentrations based on the final core mixed-effects models. Clinical variables shown were retained in the final models based on relative model fit (AIC). Points represent estimated marginal means with 95% confidence intervals (error bars) for ELISA (blue) and Luminex (orange), stratified by respiratory hospital admission, antibiotic therapy, and autoimmunity. Values are back-transformed from the log10 scale and shown in µg/mL. Predicted values are adjusted for all other fixed effects in the model and include random intercepts for individual participants

Predicted pneumococcal IgG concentrations from the final mixed-effects models are presented in Fig. 2. These models were selected based on relative model fit (AIC) and are intended to illustrate the direction and magnitude of the estimated effects rather than provide evidence of clinically meaningful group differences. Differences between groups were modest on the log10 scale and broadly consistent across assays, with largely overlapping confidence intervals.

### Interaction analyses

Exploratory interaction analyses were performed using AIC-based procedures (see Methods). No interaction term improved model fit in the ELISA- and Luminex-based models.

### Linear regression analysis of the last available measurement

**Fig. 3:**
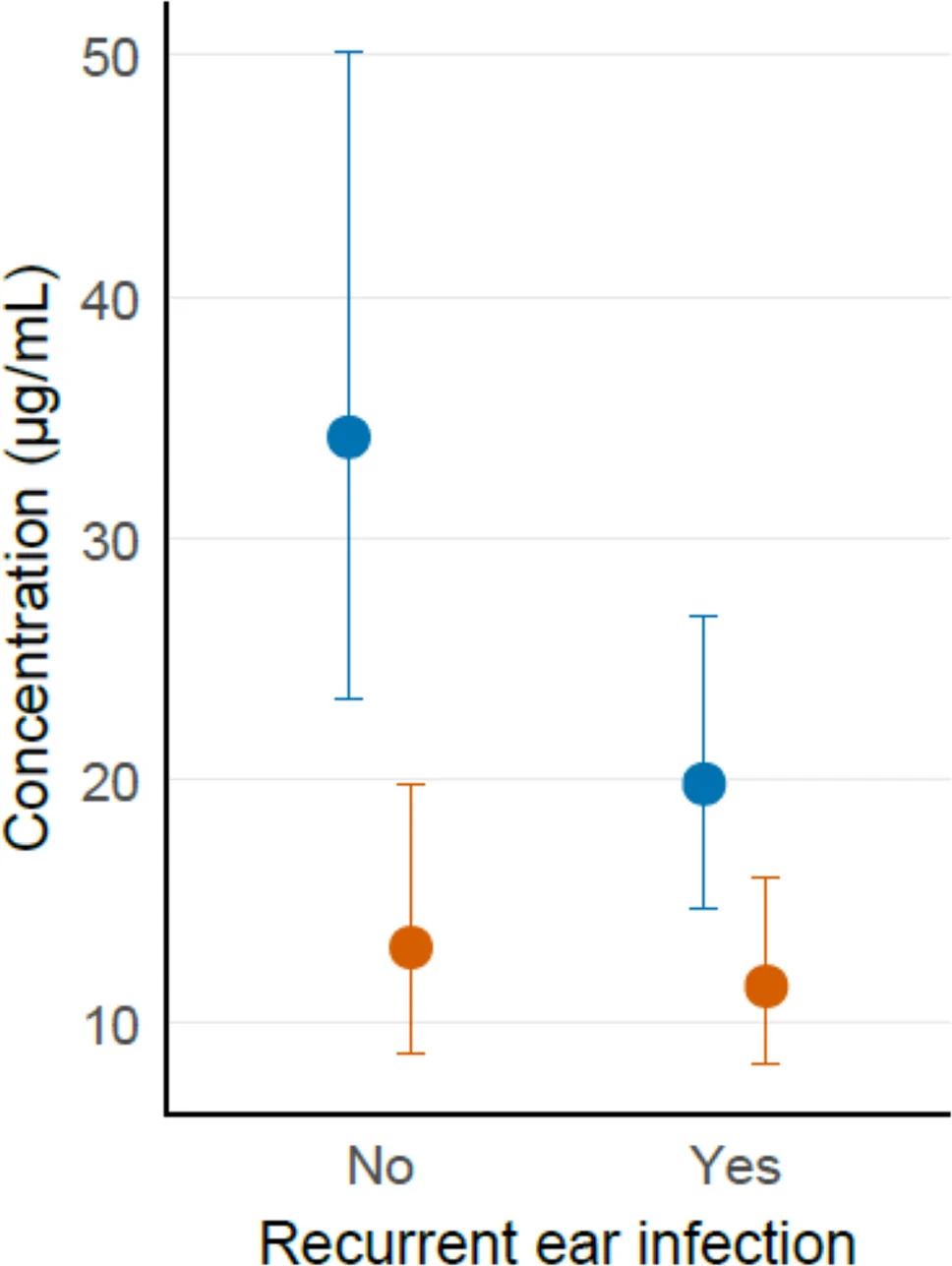
Predicted pneumococcal IgG concentration at the last available timepoint per participant, stratified by recurrent ear infection. Points represent model-based predicted means with 95% confidence intervals (error bars) from linear regression models fitted separately for ELISA (blue) and Luminex (orange). Values are shown on the original concentration scale (µg/mL). Estimates are adjusted for all other clinical variables included in the model

As a complementary last-timepoint analysis to explore the potential impact of the mismatch between static clinical variables and longitudinal antibody measurements, we performed cross-sectional linear regression on the last available measurement per participant for both assays. In the ELISA analysis, only recurrent ear infection was associated with lower log10-transformed concentrations (estimate =  − 0.24, *p* = 0.034) (Fig. 3). In the Luminex analysis, no clinical variables showed a clear association with the last available measurement, including recurrent ear infection (estimate =  − 0.06, *p* = 0.629).

Results from the single timepoint analyses were directionally consistent with the longitudinal mixed-effects models. Detailed results of the longitudinal age analyses, including effect estimates, model fit comparisons, and random intercept versus random slope comparisons, are presented in Supplementary Tables 2, 3, and 4. These analyses showed that incorporating longitudinal age structure did not materially alter the direction or magnitude of the clinical effect estimates and did not improve overall model fit.

## Discussion and conclusions

The aim of this study was to explore whether pneumococcal IgG concentrations could be related to clinical characteristics in children with DS, a population known to be at increased risk for recurrent and severe RTIs and characterised by a heterogeneous immune phenotype [4, 23]. Within this framework, pneumococcal IgG measurements were evaluated not as an endpoint in themselves, but as exploratory immunological markers that might relate to clinical heterogeneity in children with DS.

Across both ELISA- and Luminex-based longitudinal analyses, a trend towards lower pneumococcal antibody concentrations was consistently observed in children with a history of respiratory hospital admissions. This finding is biologically plausible and consistent with existing literature describing increased respiratory morbidity and hospitalisation rates in children with DS, which have been linked to altered innate and adaptive immunity [4]. Although direct evidence connecting pneumococcal antibody levels to hospitalisation risk in DS remains limited, our findings suggest that lower pneumococcal antibody concentrations may relate to a subgroup of children with more severe respiratory disease rather than merely reflecting exposure history.

The consistent direction of observed associations across both ELISA and Luminex assays suggests similar findings across platforms, despite some differences in effect size. From a practical perspective, ELISA is easier to perform in routine clinical laboratories. In the present study, we evaluated cumulative pneumococcal IgG measurements rather than individual serotype concentrations. Our results indicate that both assay platforms may capture information that could be clinically relevant related to respiratory disease severity in children with DS. However, whether additional serotype-specific resolution could provide additional clinical or immunological insight warrants further investigation.

Besides, our findings suggest that higher pneumococcal IgG concentrations should not automatically be interpreted as evidence of effective immune protection. Autoimmunity, defined by the presence of autoimmune comorbidity, improved model fit in the exploratory analyses and was associated with a positive effect estimate. Although the direction of the observed association was similar across assays, differences in effect size were observed between the platforms. This difference may reflect methodological variation between ELISA, which measures aggregated 23-valent IgG, and Luminex-based assays that quantify and sum individual serotype-specific responses. Differences in epitope presentation, assay dynamic range, or multiplex detection characteristics may contribute to this variation, and caution is warranted when directly comparing absolute effect sizes across platforms.

Autoimmune conditions are increasingly recognised as situations in which antibody production may be quantitatively preserved or elevated despite impaired protective immunity. Cunningham-Rundles (2011) describes this phenomenon, highlighting that chronic immune activation can coexist with persistent susceptibility to infection [24]. This interpretation is supported by data from a DS-specific cohort described by Musolino et al. (2025) [25]. Evidence from autoimmune disease populations further reinforces that measurable antibody concentrations do not consistently predict clinical protection [15, 26, 27]. Together, these findings underscore that pneumococcal antibody concentrations in DS should be interpreted with caution.

Several potential clinical triggers for pneumococcal antibody testing, such as recurrent ear infections or frequent upper RTIs, did not consistently align with repeated antibody levels in our mixed-effects models. This observation probably reflects the multifactorial nature of recurrent infections in children with DS, which often arise from a combination of anatomical factors, airway clearance abnormalities, and non–antibody-mediated immune mechanisms, potentially decoupling infection frequency from humoral responses [4].

An important challenge in interpreting the longitudinal analysis was that the clinical variables were not recorded longitudinally, whereas pneumococcal IgG concentrations were measured repeatedly over time. This limitation was inherent to the retrospective study design, which restricted the temporal precision and completeness of the available clinical data. To partly address this mismatch, we performed a cross-sectional analysis using only the last available pneumococcal IgG measurement per participant. This approach reduced, but did not eliminate, the likelihood that antibody measurements preceded the recorded clinical characteristics, although the interval between clinical events and serological measurement could still be substantial. In this analysis, recurrent ear infection was associated with lower ELISA-measured IgG concentrations, a finding not observed in the longitudinal models or in the Luminex analyses. This discrepancy illustrates how temporal alignment can influence observed associations and reinforces the importance of longitudinal approaches when interpreting pneumococcal serology. In addition, the relatively modest cohort size reduced statistical power, particularly for interaction analyses and less common clinical characteristics. Although missing data were generally limited, incomplete availability of some clinical variables may also have influenced individual exploratory analyses.

Current health supervision guidelines for children with DS do not recommend routine immunological testing but advise further immune evaluation in the presence of recurrent or severe infections [28, 29]. The decision to start antibiotic prophylaxis remains clinical evaluation. Our explorations also do not support routine pneumococcal serology in all children with DS, however, they do suggest that further research into the potential clinical relevance of pneumococcal antibody measurements in children with DS may be warranted, particularly in those children with more severe respiratory disease.

## Supplementary information

Below is the link to the electronic supplementary material.

ESM 1 (DOCX 209 KB)

## Data Availability

The datasets generated and/or analysed during the current study are not publicly available but are available from the corresponding author on reasonable request.

## Abbreviations

AIC: Akaike information criterion
CPS: Cell wall polysaccharide
DS: Down syndrome
EHR(s): Electronic health record(s)
ETZ: Elisabeth-Tweesteden Hospital
GLM: Generalised linear modelling
LM: Linear modelling
LMER: Linear mixed effects modelling
LMMI: Laboratory for Medical Microbiology and Immunology
MMC: Máxima Medical Centre
PnP: Pneumococcal polysaccharide
RTI(s): Respiratory tract infection(s)
UTI(s): Urinary tract infection(s)
