# Supplementary material for: Lower pneumococcal IgG levels and respiratory hospitalisation in children with Down syndrome; an exploratory study
Source: Eur J Pediatr. 2026 Aug 28;185(9):699. doi: 10.1007/s00431-026-07364-w (PMC13524981; doi:10.1007/s00431-026-07364-w)
Supplement: Supplementary file 1 [file 431_2026_7364_MOESM1_ESM.docx]

# Online Resource (Supplementary figures)

**Supplementary figure 1**. Scatterplot of log10-transformed pneumococcal IgG concentrations measured by ELISA and Luminex. Grey circles represent samples included in the final analysis. The dashed line indicates the linear regression fit between assays. The black diamond denotes the sample identified as a consistent two-dimensional (2D) outlier based on discordance between ELISA and Luminex measurements and excluded from both assays in subsequent analyses. The black triangle represents a Luminex-specific one-dimensional (1D) outlier. No ELISA-specific 1D outliers were identified.

**Supplementary figure 2.** Histograms of raw and log10-transformed pneumococcal IgG concentrations for ELISA and Luminex assays. Raw concentrations exhibited right-skewed distributions. Log₁₀ transformation improved distributional symmetry and was therefore applied in all regression and mixed-effects models.

**Supplementary figure 3.** Collinearity was assessed using Spearman correlation analyses between candidate predictors, age at sampling, and vaccination status. The strongest correlation was observed between age at sampling and vaccination status (r = -0.69). To avoid multicollinearity and facilitate interpretation, age and vaccination status were not included simultaneously in the primary regression models.

**Supplementary Table 1. An overview of missing data per variable.**

| **Variable** | **Available n** | **Missing n** |
| --- | --- | --- |
| **Respiratory hospital admission** | 52 | 10 |
| **Autoimmunity** | 62 | 0 |
| **Therapeutic antibiotic use** | 53 | 9 |
| **Antibiotic prophylaxis use** | 62 | 0 |
| **Recurrent ear infections** | 62 | 0 |
| **Infections in past year** | 62 | 0 |
| **Inhalation medication** | 62 | 0 |
| **Congenital heart disease** | 62 | 0 |

**Supplementary Table 2. Comparison of clinical effect estimates before and after inclusion of longitudinal age variables.**

| **Variable** | **ELISA no age β (95% CI)** | **ELISA + age RI β (95% CI)** | **Luminex no age β (95% CI)** | **Luminex + age RI β (95% CI)** |
| --- | --- | --- | --- | --- |
| **Recurrent ear infections** | -0.13 (-0.34, 0.08) | -0.13 (-0.33, 0.08) | 0.03 (-0.21, 0.28) | 0.02 (-0.21, 0.25) |
| **Respiratory admission** | -0.04 (-0.26, 0.19) | -0.03 (-0.25, 0.20) | -0.01 (-0.27, 0.25) | 0.01 (-0.24, 0.25) |
| **Antibiotic prophylaxis** | -0.05 (-0.29, 0.19) | -0.01 (-0.26, 0.23) | -0.09 (-0.38, 0.20) | 0.00 (-0.29, 0.29) |
| **Therapeutic antibiotics** | -0.08 (-0.34, 0.18) | -0.11 (-0.37, 0.15) | -0.02 (-0.33, 0.30) | -0.08 (-0.38, 0.22) |
| **Immune dysregulation** | 0.20 (-0.00, 0.41) | 0.19 (-0.02, 0.39) | 0.18 (-0.06, 0.42) | 0.16 (-0.07, 0.38) |
| **Inhalation medication** | 0.15 (-0.12, 0.42) | 0.14 (-0.13, 0.41) | 0.02 (-0.30, 0.34) | -0.01 (-0.32, 0.29) |
| **Infection past year** | -0.12 (-0.34, 0.10) | -0.10 (-0.32, 0.12) | -0.13 (-0.39, 0.13) | -0.12 (-0.35, 0.14) |
| **Sex** | 0.01 (-0.21, 0.24) | 0.02 (-0.21, 0.24) | 0.07 (-0.20, 0.35) | 0.06 (-0.20, 0.31) |
| **Congenital heart disease** | 0.06 (-0.16, 0.28) | 0.10 (-0.13, 0.32) | 0.04 (-0.22, 0.29) | 0.10 (-0.15, 0.35) |

Estimates represent fixed-effect coefficients (β) from linear mixed-effects models. Age-adjusted models included age at first sample and age increase since the previous sample as fixed effects and a participant-specific random intercept.

Inclusion of longitudinal age variables did not materially alter the magnitude or direction of the clinical effect estimates.

**Supplementary Table 3. Comparison of model fit with and without longitudinal age structure.**

| **Assay** | **Model** | **AIC** |
| --- | --- | --- |
| **ELISA** | Original clinical model | 137.99 |
| **ELISA** | + longitudinal age (RI) | 162.42 |
| **ELISA** | + longitudinal age (RS) | 160.47 |
| **Luminex** | Original clinical model | 122.53 |
| **Luminex** | + longitudinal age (RI) | 138.01 |
| **Luminex** | + longitudinal age (RS) | 139.66 |

Lower AIC values indicate better model fit. Age-adjusted models included age at first sample and age increase since the previous sample as fixed effects. RI = random intercept model; RS = random slope model. Models without longitudinal age variables showed the lowest AIC values for both assays.

**Supplementary Table 4. Comparison of random intercept and random slope age models.**

| **Assay** | **RI AIC** | **RS AIC** | **Likelihood ratio χ²** | **df** | **p-value** |
| --- | --- | --- | --- | --- | --- |
| **ELISA** | 108.51 | 107.19 | 5.32 | 2 | 0.070 |
| **Luminex** | 86.34 | 88.42 | 1.92 | 2 | 0.383 |

RI models included a participant-specific random intercept only. RS models additionally included a random slope for age increase since the previous sample. Likelihood ratio tests were used to compare RI and RS models. Addition of a random slope for within-participant age increase did not meaningfully improve model performance and was therefore not retained in the final models.

**Supplementary Table 5. Number of samples per participant**

| **Number of samples** | **Number of participants** |
| --- | --- |
| **1** | 11 |
| **2** | 14 |
| **3** | 8 |
| **4** | 13 |
| **5** | 7 |
| **6** | 8 |
| **7** | 1 |
